# Sequencing and analysis of the complete mitochondrial genome of *Micromys erythrotis* from China and its phylogenetic analysis

**DOI:** 10.1080/23802359.2021.1926353

**Published:** 2021-05-12

**Authors:** He Cai, Qing-Qing Wang, Xin-Xu Zhao, Qian-Qian Yao, Ning Wu, Jun-Sheng Zhang, Zhu Liu

**Affiliations:** a College of Life Science and Technology, Mudanjiang Normal University, Mudanjiang, P.R. China; b Ningan Jiangnan School, Ningan, P.R. China

**Keywords:** Control region, mitogenome, phylogenetic trees, *Micromys erythrotis*

## Abstract

The complete mitogenome sequence of *Micromys erythrotis* was determined using long PCR. The genome was 16,238 bp in length and contained 13 protein-coding genes, 2 ribosomal RNA genes, 22 transfer RNA genes, 1 origin of L strand replication and 1 control region. The overall base composition of the heavy strand is A (33.7%), C (24.8%), T (29.1%) and G (12.4%). The base compositions present clearly the A–T skew, which is most obviously in the control region and protein-coding genes. Mitochondrial genome analyses based on MP, ML, NJ and Bayesian analyses yielded identical phylogenetic trees. This study verifies the evolutionary status of *Micromys erythrotis* in Muridae at the molecular level. The mitochondrial genome would be a significant supplement for the *Micromys erythrotis* genetic background.

The existence of a second species of *Micromys* is considered by Wroughton (1920) and Yasuda et al.(2005). *Micromys erythrotis* is considered to be an independent species, through morphological and molecular biology research (Abramov et al. 2009). In this paperA muscle sample was obtained from a female *Micromys erythrotis* captured from Bijie regions of Wumeng Mountains in Guizhou Province, China (26°24′22″ N,105°44′04″ E). The muscle tissue was preserved in 95% ethanol and stored at −75 °C before use. The specimen and its DNA is stored in Animal and Plant Herbarium of Mudanjiang Normal University. The voucher number is HECS2019014. Genomic DNA was extracted from muscle using the EasyPure genomic DNA kit (TransGen Biotech Co., Beijing, China). The mitogenomes were sequencing by Illumina NovaSeq 6000 platform (Ruiboxingke Biotechnology Co. Ltd., Beijing, China) using a primer walking strategy and the long and accurate PCR. The draft sequence was manually corrected. The complete mitochondrial genome sequence was annotated using Sequin.

The mitochondrial genome is a circular double-stranded DNA sequence that is 16,238 bp long including 13 protein-coding genes, 2 rRNA genes, 22 tRNA genes, 1 origin of L strand replication and 1 control region. The accurate annotated mitochondrial genome sequence was submitted to GenBank with accession number MW389539. The arrangement of the multiple genes is in line with other Muridae species (Robins et al. 2008; Chen et al. 2012; Jing et al. 2015; Chang et al. 2016; Yong et al. 2016; Zhang et al. 2016; Wei et al. 2017; Lv et al. 2019) and most mammals (Mouchaty et al. 2000; Nikaido et al. 2001; Nikaido et al. 2003; Fontanillas et al. 2005; Cabria et al. 2006; Meganathan et al. 2012; Yoon et al. 2013; Xu et al. 2012, 2013; Kim et al. 2013, 2017; Hou et al. 2016; Huang et al. 2014, 2016; Xu et al. 2016; Liu et al. 2016; Liu, Tian, Jin, Jin, et al. 2017; Liu, Tian, Jin, Dong, et al. 2017; Liu, Wang, et al. 2017; Liu et al. 2018; Liu, Dang, et al. 2019; Liu, Qin, et al. 2019; Jin et al. 2017; Gutiérrez et al. 2018; Jia et al. 2018).

The control region of *Micromys erythrotis* mitochondrial genome was located between the tRNA-Pro and tRNA-Phe genes, and contains only promoters and regulatory sequences for replication and transcription, but no structural genes. Three domains were defined in *Micromys erythrotis* mitochondrial genome control region (Zhang et al., 2009): the extended termination-associated sequence (ETAS) domain, the central conserved domain (CD) and the conserved sequence block (CSB) domain.

The total length of the protein-coding gene sequences was 11,372 bp. Most protein-coding genes initiate with ATG except for ND1, ND2 and ND3, which began with GTG or ATT. Nine protein-coding genes terminated with TAA whereas the Cyt b gene terminated with AGC. The incomplete stop codons (T– –) were used in COX3 and ND4, The others are TAG. A strong bias against A at the third codon position was observed in the protein-coding genes. The frequencies of CTA (Leu), ATT (Ile), TTA (Leu) and ATA (Met) were higher than those of other codons. The length of tRNA genes varied from 58 to 76 bp.

Most *Micromys erythrotis* mitochondrial genes were encoded on the H strand, except for the ND6 gene and eight tRNA genes, which were encoded on the L strand. Some reading frame intervals and overlaps were found. One of the most typical was between ATP8 and ATP6. The L-strand replication origin (OL) was 32 bp long and had the potential to fold into a stable stem-loop secondary structure. The total base composition of *Micromys erythrotis* mitochondrial genome was A (33.7%), C (24.8%), T (29.1%) and G (12.4%). The base compositions clearly present the A-T skew, which was most obviously in the control region and protein coding genes.

In order to explore the evolution of Muridae species which include Ochotonidae and Leporidae, especially the evolution of genus *Micromys* from China, here, we investigate the molecular phylogenetics of Chinese *Micromys erythrotis* using complete mitochondrial genome sequence of 40 species. All sequences generated in this study have been deposited in the GenBank (Figure 1).

**Figure 1.**
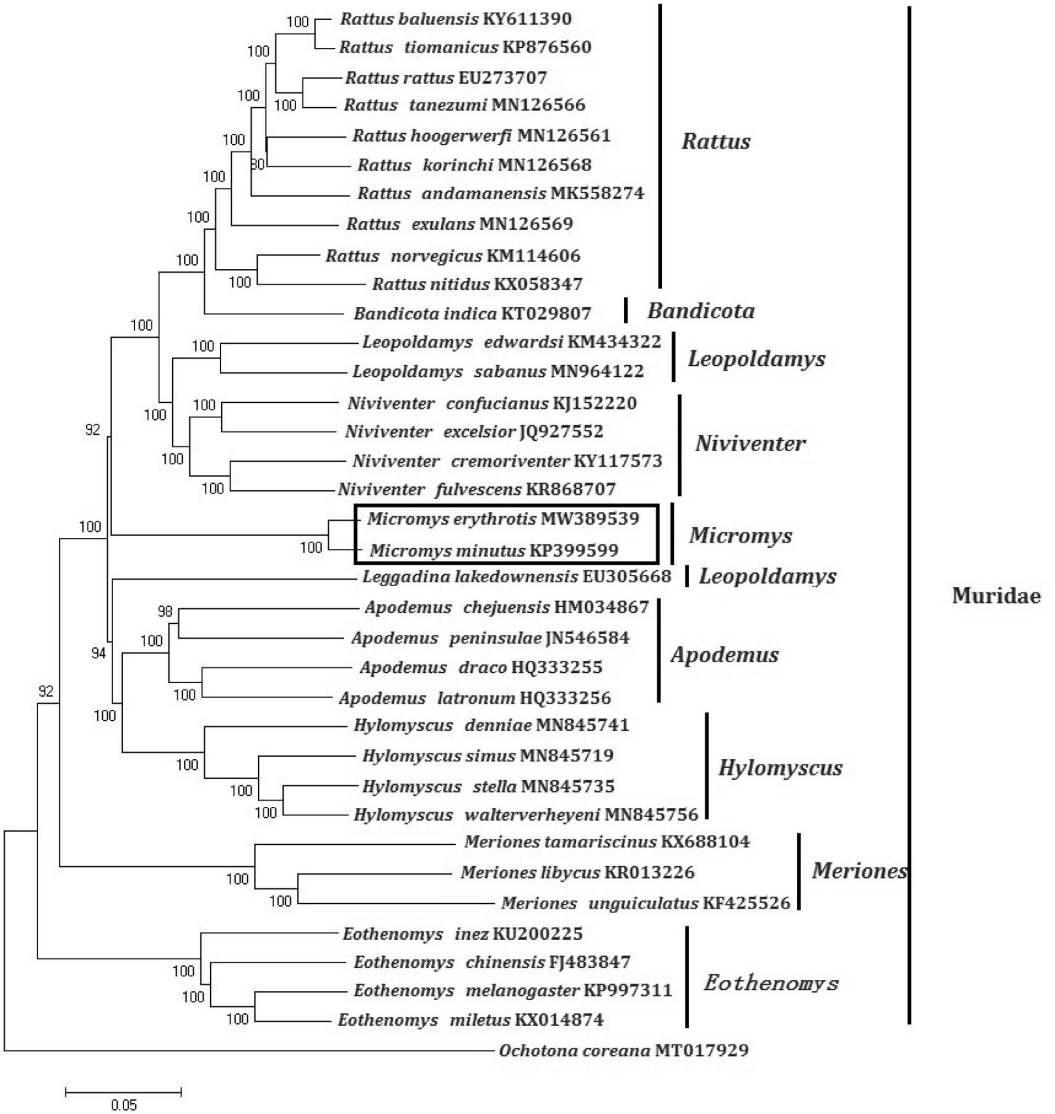
Phylogenetic tree generated using the Maximum Likelihood method based on complete mitochondrial genomes. *Rattus andamanensis* (MK558274), *Rattus nitidus* (KX058347), *Rattus rattus* (EU273707), *Rattus norvegicus* (KM114606), *Rattus tiomanicus* (KP876560), *Rattus exulans* (MN126569), *Rattus korinchi* (MN126568), *Rattus tanezumi* (MN126566), *Rattus hoogerwerfi* (MN126561), *Rattus baluensis* (KY611390), *Niviventer fulvescens* (KR868707), *Niviventer confucianus* (KJ152220), *Niviventer excelsior* (JQ927552), *Niviventer cremoriventer* (KY117573), *Bandicota indica* (KT029807), *Hylomyscus denniae* (MN845741), *Hylomyscus walterverheyeni* (MN845756), *Hylomyscus stella* (MN845735), *Hylomyscus simus* (MN845719), *Leopoldamys edwardsi* (KM434322), *Leopoldamys sabanus* (MN964122), *Apodemus peninsulae* (JN546584), *Apodemus latronum* (HQ333256), *Apodemus draco* (HQ333255), *Apodemus chejuensis* (HM034867), *Micromys minutus* (KP399599), *Micromys erythrotis* (MW389539), *Eothenomys miletus* (KX014874), *Eothenomys melanogaster* (KP997311), *Eothenomys chinensis* (FJ483847), *Eothenomys inez* (KU200225), *Leggadina lakedownensis* (EU305668), *Meriones tamariscinus* (KX688104), *Meriones libycus* (KR013226), *Meriones unguiculatus* (KF425526). The out group is *Ochotona coreana* (MT017929).

Mitochondrial genome analyses based on ML phylogenetic tree, indicating a close phylogenetic affinity of species through MEAG 5.0 software. The phylogram obtained from Maximum Parsimony method is shown in Figure 1. It shows that one major phyletic lineages were present in Muridae. In this study, the 10 genera (*Rattus*, *Niviventer*, *Bandicota*, *Hylomyscus*, *Leopoldamys*, *Apodemus*, *Micromys*, *Eothenomys*, *Leggadina*, and *Meriones*) included in Muridae form independent branches. *Micromys* comprised *Micromys erythrotis* and *Micromys minutus* was supported by bootstrap values of 100%. This study verifies the evolutionary status of *Micromys erythrotis* in Muridae at the molecular level. The mitochondrial genome would be a significant supplement for the *Micromys erythrotis* genetic background.

## Data Availability

The data that support the findings of this study are openly available in GenBank at https://www.ncbi.nlm.nih.gov/, reference number MW389539.
